# Impacts of diarrhea on the immune system, intestinal environment, and expression of PGRPs in New Zealand rabbits

**DOI:** 10.7717/peerj.4100

**Published:** 2017-11-27

**Authors:** Yang Chen, Bohao Zhao, Yuwei Wu, Shuaishuai Hu, Lin Mu, Cigen Zhu, Yulai Pan, Xinsheng Wu

**Affiliations:** 1 College of Animal Science and Technology, Yangzhou University, Yangzhou, Jiangsu, China; 2 Yangzhou University, College of Veterinary Medicine, Yangzhou, Jiangsu, China; 3 Jinling Rabbit Farm, Nanjing, Jiangsu, China

**Keywords:** Intestinal environment, Peptidoglycan recognition proteins, Microbiome, Diarrhea, New Zealand rabbit

## Abstract

Diarrhea is a syndrome of digestive disorders in young rabbits and may lead to secondary infections resulting in reduced immunity and higher mortality in baby rabbits, with serious impacts on rabbit farming. In this study, we investigated the effects of diarrhea on the health of baby rabbits in terms of intestinal mucosal development, immune function, and intestinal microbial diversity. We found that the duodenal villus length and the villus/crypt ratio in rabbits with diarrhea were significantly reduced compared with those in healthy rabbits (*P* < 0.01). Rabbits with diarrhea had significantly lower concentrations of acetic acid (*P* < 0.05), higher pH levels (*P* < 0.05), and higher levels of ammonia nitrogen (*P* < 0.01) in the cecum. Moreover, diarrhea in baby rabbits led to significantly reduced levels of total serum protein (*P* < 0.05) and markedly increased levels of alkaline phosphatase, urea nitrogen, TNF-*α*, and IL-6 (*P* < 0.05). Transcriptional analysis of peptidoglycan recognition proteins (PGRPs, including *PGLYRP-1*, *PGLYRP-2*, and *PGLYRP-3*) using real-time PCR revealed that diarrhea induced the upregulation of PGRPs in the cecum and duodenum. Furthermore, through pyrosequencing of the 16S rRNA V4 region in cecum samples, we found that the total number and diversity of microbes were not significantly different between healthy rabbits and those with diarrhea, though there were noticeable differences in the prevalences of *Clostridium*, *Roseburia,* and *Alistipes*. Our results will contribute to a better understanding of the pathological mechanisms of diarrhea in young rabbits.

## Introduction

Diarrhea is a common disease in rabbits, especially in weaned baby rabbits, in which it has the highest incidence among all diseases (Beltz, Rosales & Morales, 2005). It causes a disturbance in the intestinal microflora and sometimes death, resulting in considerable losses to the rabbit farming industry (Pascual, 2001). Therefore, we analyzed the effects of diarrhea on the intestinal development and environment in weaned rabbits and characterized the intestinal flora associated with diarrhea in rabbits, in order to elucidate the association between the rabbit intestinal environment and its health.

A normal intestinal flora is important for the maintenance of animal health in terms of intestinal development, nutrient digestion and absorption, and immunity (Guarner & Malagelada, 2003). Thinning of the intestinal wall, shortening of the intestinal tract, and shrinking of intestinal villi are detrimental to intestinal development and nutrient absorption (Falk et al., 1998). There are 10^9^–10^10^ *Bacteroides* in every gram of cecal content in an adult rabbit, whereas the total amount of *Bifidobacteria*, *Clostridium*, *Streptococcus*, and *Enterobacter* is 10^10^–10^12^ CFU/g (De Blas & Wiseman, 2010). These microorganisms participate in the metabolism of urea, ammonia, and peptides, as well as in the hydrolysis of fibers by secreting digestive enzymes or through their own metabolism, producing volatile fatty acids (VFA), amino acids, and vitamins. They thereby play a crucial role in host immunity and the modulation of the host intestinal microenvironment (Aerts, Boever & Maertens, 2010; Macfarlane, Hopkins & Macfarlane, 2000). Peptidoglycan recognition proteins (PGRPs), which are highly conserved in insects and mammals, are a group of innate immunity-activating molecules (Kang et al., 1998; Liu et al., 2001). The sequences of *PGLYRP-1*, *PGLYRP-2*, and *PGLYRP-3* have been determined through homology searching of the NCBI and Ensembl databases, yet their functions in rabbit diarrhea have not been documented.

In this study, intestinal tissues and blood samples were collected from rabbits with diarrhea and healthy rabbits for analysis of changes in intestinal histology, serum immune indicators, and the expression of PGRPs in rabbits with diarrhea. Moreover, 16S rDNA amplicon sequencing was performed to compare bacterial amounts and distributions between rabbits with diarrhea and healthy rabbits at the DNA level. Our study comprehensively demonstrates the alterations in the intestinal environment accompanying diarrhea and provides theoretical evidence for the prevention of diarrhea in weaned rabbits.

## Materials and Methods

### Ethics statements

All animal experiments used in this study were approved by the Institutional Animal Care and Use Committee of Yangzhou University (Jiangsu, China) and were strictly implemented according to the regulations for experimental animals. An ordinary housing facility was used and was in keeping with the national standard Laboratory Animal Requirements of Environment and Housing Facilities (GB 14925-2001). The care of laboratory animals and the animal experimental operations conformed to the Jiangsu Administration Rule of Laboratory Animal. The rabbits were anesthetized by an intraperitoneal injection of sodium pentobarbital.

### Animals and sampling

New Zealand rabbits weaned at 35 days of age were used as experimental animals to study the impacts of diarrhea. Rabbits with spontaneous diarrhea were collected 10 days after weaning. Moreover, eight weaned rabbits with diarrhea and eight healthy weaned rabbits were obtained and sacrificed. The cecal contents of each rabbit were collected in three 2-mL cryotubes and stored in liquid nitrogen for later determination of VFA and ammonia nitrogen (NH_3_-N) levels, as well as 16S high-throughput sequencing. The duodenum and cecum samples were collected at the same position in each rabbit and stored in liquid nitrogen for later RNA extraction. A fragment of the duodenum was removed at the same position, rinsed with 0.9% saline, and fixed in paraformaldehyde for the preparation of intestinal sections.

### Assessment of intestinal environment

Cecal contents were collected, and the pH was measured immediately with a pH meter (DELTA 320, Shanghai, China). Duodenal contents (2 mL) were mixed with 2 mL distilled water and recovered by centrifugation at 10,000 rpm for 10 min. Subsequently, 1 mL supernatant was collected and mixed with 0.2 mL 20% metaphosphoric acid solution containing 60 mM crotonic acid. VFA levels were determined using a GC-9A Gas Chromatograph (Shimadzu, Japan) with nitrogen as the carrier gas, a flow-rate of 30 mL/min. A CP-WAX capillary column with a length of 30 m, an inner diameter of 0.53 mm, and a membrane thickness of 1 µm was used, and the sample input was 0.6 µL.

Diluted cecal contents (4 mL) were centrifuged for 10 min, and 50 µL supernatant was collected in a 10-mL tube. Phenol and sodium hypochlorite (3 mL each) were added to the supernatant, and the mixture was incubated in a 60 °C water bath for 10 min, followed by immediate cooling. The optical density (OD) at 546 nm was measured, and the concentration of NH_3_-N in the cecum was analyzed based on a standard curve of ammonium chloride.

### Determination of serum indicators

Blood was drawn from rabbits with diarrhea and healthy rabbits, allowed to settle for 30 min, and centrifuged at 3,500 rpm for 15 min to isolate serum. Serum indicators were detected according to the instructions of the manufacturer (Nanjing Jiancheng Bioengineering Institute, Nanjing, China). The factors used to evaluate the serum biochemical profile included serum glucose (GLU), total protein (TP), urea nitrogen (UN), and serum alkaline phosphatase (AKP). In particular, serum immune indicators, including interleukin-2 (IL-2), interleukin-6 (IL-6), tumor necrosis factor-*α* (TNF-*α*), immunoglobulin G (IgG), and immunoglobulin M (IgM), reflected the immune response to diarrhea.

The level of GLU was measured using the glucose oxidase method. TP content in the serum was determined by the biuret method. The level of UN in the serum was determined using the urease method. AKP was measured by colorimetry. The serum levels of IL-2, IL-6, TNF-*α*, IgG, and IgM were determined by double-antibody sandwich enzyme-linked immunosorbent assay (ELISA).

### Measurement of intestinal villus length and crypt depth

**Figure 1:**
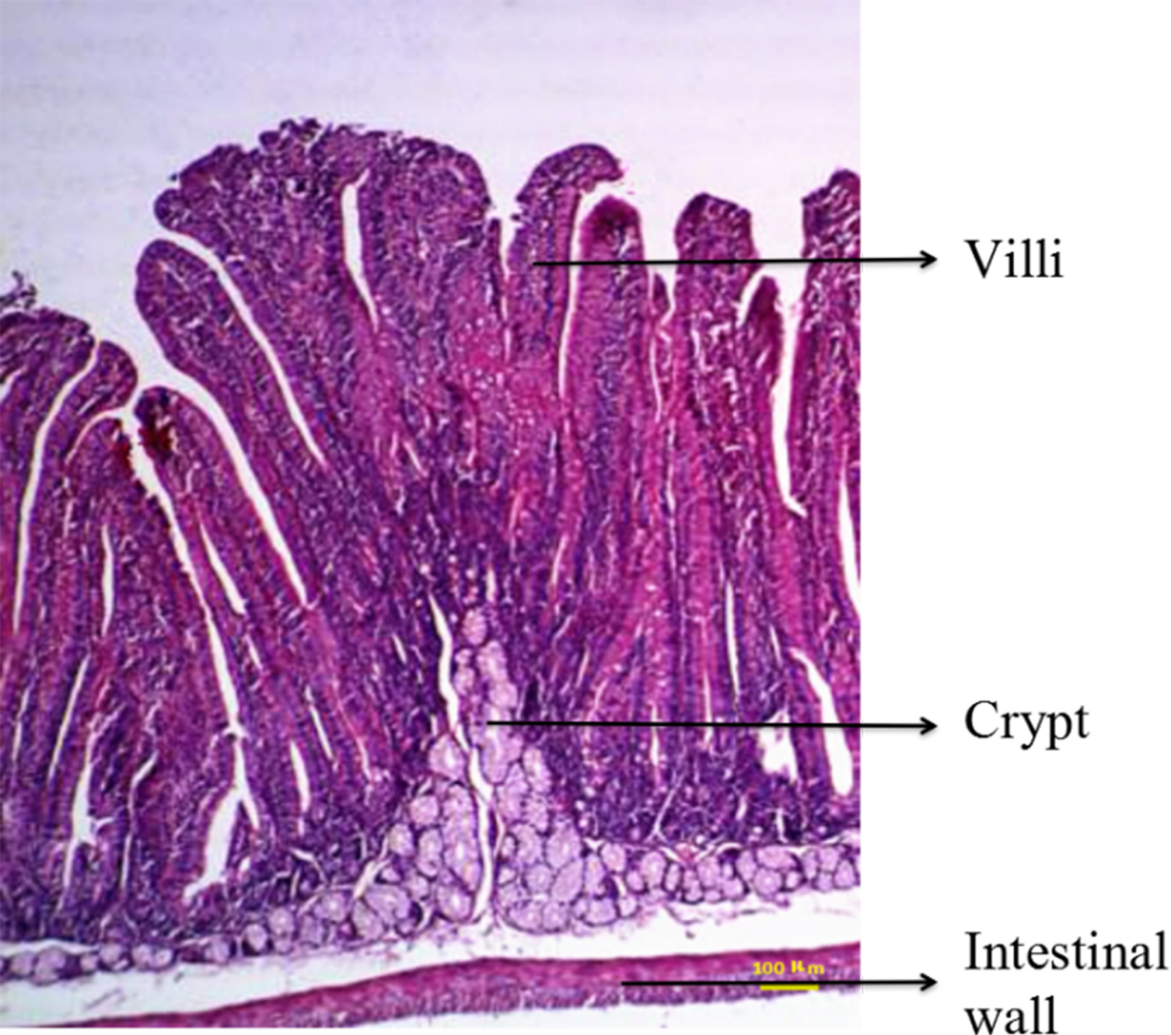
Duodenal structure of weaned rabbits (HE staining, 100×).

The intestine was examined by routine HE staining of tissue paraffin sections. Five consecutive longitudinal sections were obtained from each segment of the intestinal tract, and six typical microscopic fields were assessed for each tissue section. The lengths of the longest villus and the corresponding crypt in each field were measured. The intestinal wall, villi, and crypt are shown in Fig. 1. The mean values for villus length and crypt depth were calculated.

### Real-time PCR

Real-time PCR was carried out on Applied Biosystems 7500 Real-Time RT-PCR System with the following program: 95 °C for 30 s, followed by 40 cycles of 95 °C for 5 s and 60 °C for 34 s. All cDNA samples were tested three times, and the results were normalized to the levels of *GAPDH* expression using the SYBR Premix Ex Taq II system (Takara, Japan). Based on the mRNA sequences of rabbit PGRPs published in GenBank, real-time PCR primers for *PGLYRP-1, PGLYRP-2*, and *PGLYRP-3* were designed and are shown in Table 1. The following equation was used to obtain the fold change: $RQ= \frac{(1+{E}_{a})^{C{T}_{ca}-C{T}_{ta}}}{(1+{E}_{k})^{C{T}_{ck}-C{T}_{tk}}} $, where *E*_*a*_ is the amplification efficiency of the target gene, *E*_*k*_ is the amplification efficiency of the internal control gene, *CT*_*ca*_ is the *C*_*t*_ value of the target gene in the control group, *CT*_*ta*_ is the *C*_*t*_ value of the target gene in the test group, *CT*_*ck*_ is the *C*_*t*_ value of the internal control gene in the control group, and *CT*_*tk*_ is the *C*_*t*_ value of the internal control gene in the test group.

**Table 1:** Real-time PCR primer sequences.

Gene	Primer sequences (5′ → 3′)	Product length (bp)
GAPDH	F: GACTTCAACAGTGCCACC	112
	R: TGCTGTAGCCAAATTCGT	
PGLYRP-1	F: CTGCTGCCTGCCGTGTGCGA	115
	R: CTGAAGACCCCAGCCCGGAG	
PGLYRP-2	F: ACTCCGTGGCTTCCTCCTG	123
	R: TGCCCGTCCCGTACATCA	
PGLYRP-3	F: ATTTCGTAGAGCTGGTTGCC	113
	R: CAGAGCCTGCTGGACTGC	

### Statistical analysis

Each experiment was repeated at least three times, and the data were analyzed using *t*-tests for independent samples and one-way ANOVA (SPSS version 21). The results are presented as the mean ± SD at two levels of significance, *P* < 0.05 and *P* < 0.01.

### 16S rDNA amplicon pyrosequencing

Homogenized cecal contents weighing 3 g (wet weight) from each healthy rabbit and rabbit with diarrhea were used (three rabbits from each group) for DNA extraction. Genomic DNA was extracted using hexadecyl trimethyl ammonium bromide (CTAB). After testing for DNA purity and concentration, an appropriate amount of sample was diluted with sterile water to a final concentration of 1 ng/µL.

The 16S V4 amplicon was amplified by PCR using specific barcode-containing primers (515F 5′-GTGCCAGCMGCCGCGGTAA-3′, 806R 5′-GGACTACHVGGGTWTCTAAT-3′). The PCR products were mixed in equal quantities based on their concentrations, and the mixture was subjected to electrophoresis on a 2% agarose gel (1 × TAE) for the purification of PCR products. The major bands at 400–450 bp were collected using GeneJET (Thermo Scientific). Thereafter, the library was constructed using the TruSeq DNA PCR-Free Library Preparation Kit (Illumina, San Diego, CA, USA), and the quality of the library was assessed using a Qubit® 2.0 Fluorometer and Agilent Bioanalyzer 2100 system. After quality testing, libraries were sequenced by Illumina HiSeq.

### Sequence analysis

We merged paired-end reads from the original DNA fragments and applied quality filters using FLASH (Magoc & Salzberg, 2011). The resulting reads were analyzed by assignment to each sample based on their unique barcodes. We processed the sequences using the QIIME (Quantitative Insights Into Microbial Ecology) software package. The alpha- and beta- diversity were parsed by in-house Perl scripts (Caporaso et al., 2010) to assess diversity within and among samples, respectively. The pick_de_novo_otus.py workflow was used to select operational taxonomic units (OTUs) after applying QIIME quality filters. Sequences with >97% similarity were defined as the same OTU. Taxonomic classifications for representative sequences were determined used the Ribosomal Database Project (RDP) classifier (Wang et al., 2007b). We used principal components analysis (PCA) to examine differences between samples along two axes and visualize them (Avershina, Frisli & Rudi, 2013). To further investigate differences in microbial diversity among the samples, statistical tests were conducted using MetaStat. The 16S rDNA sequences were deposited with the NCBI (accession number: SRP114741).

## Results

### Comparison of intestinal morphology and environment between rabbits with diarrhea and healthy rabbits

**Figure 2:**
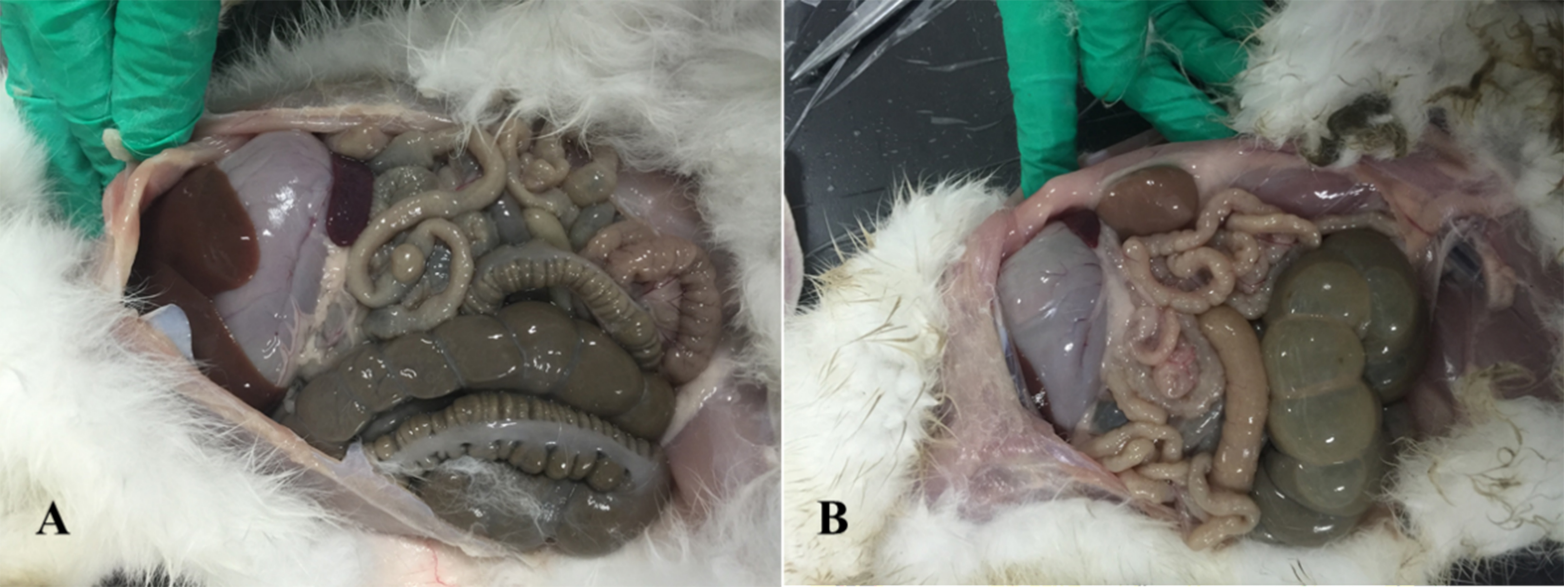
Intestinal changes in rabbits with diarrhea. (A) Healthy rabbits (*n* = 8). (B) Rabbits with diarrhea (*n* = 8), presenting with severe flatulence, wall thinning, and watery contents in the cecum and rectum.

**Figure 3:**
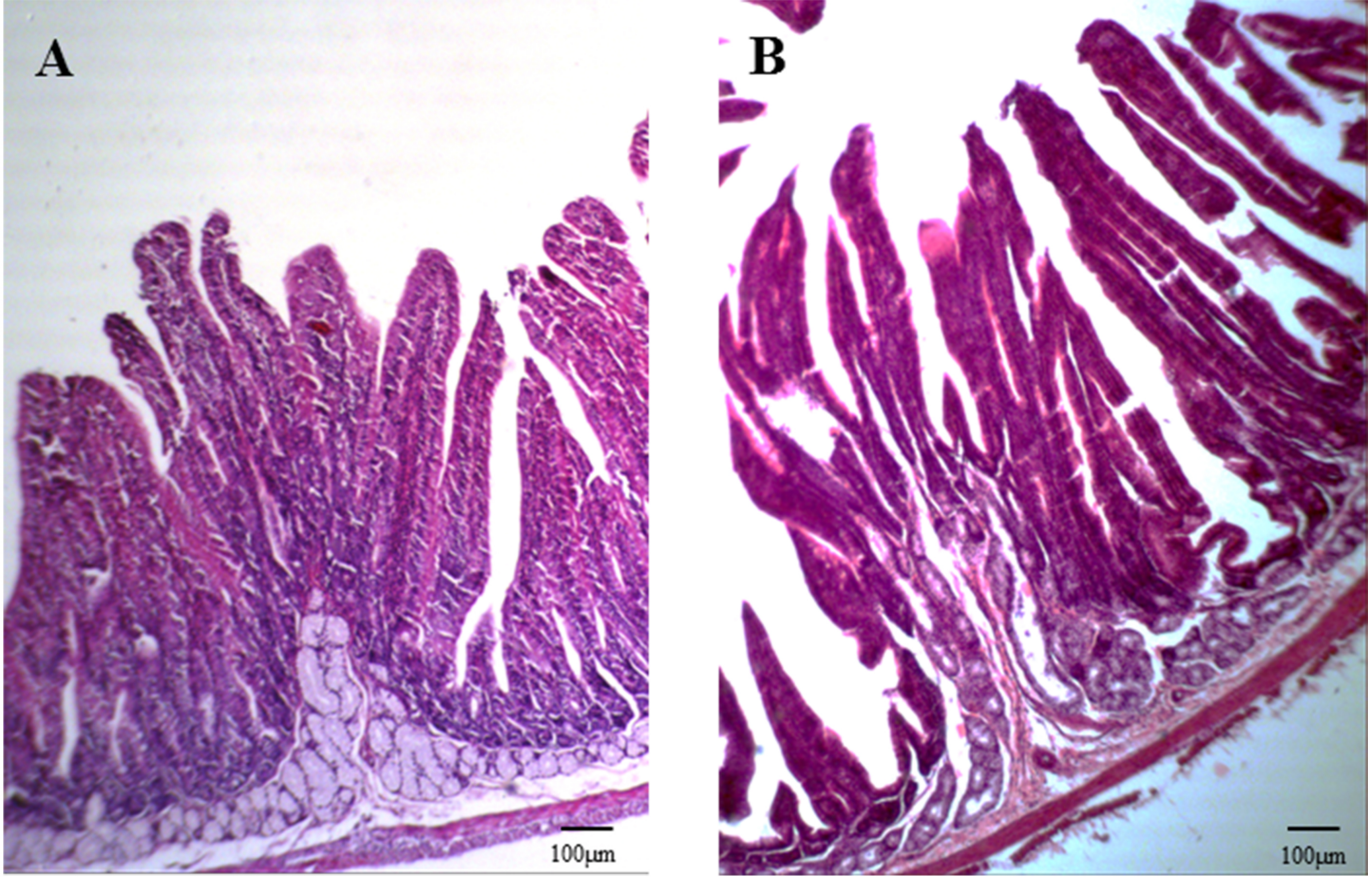
Duodenal section from a healthy rabbit and rabbit with diarrhea (HE staining, 100×). The intestinal wall, villi, and crypt of the duodenum of three weaned rabbits with diarrhea and three healthy weaned rabbits were measured.

Anatomical examination showed serious flatulence in the ceca and recta of young rabbits with diarrhea, accompanied by thinning of the cecal and rectal walls and watery contents. Figure 2 shows the duodenal morphology and cecal environment of the rabbits. Histological analysis revealed that the density of duodenal villi in rabbits with diarrhea was lower than that in the healthy rabbits, and the morphology of the diarrheal duodenum was not intact (Fig. 3). Statistical analysis indicated that, compared with the healthy rabbits, rabbits with diarrhea had markedly shorter villous lengths and smaller villus/crypt ratios (*P* < 0.01), as well as significantly thinner duodenal walls (*P* < 0.05). However, crypt depth was not significantly different between the two groups (Table 2). These results indicate that diarrhea leads to severe damage to the duodenal structure in rabbits.

**Table 2:** Comparison of duodenal morphology between healthy rabbits and those with diarrhea.

Variable	Healthy	Diarrhea
Villus, mm	0.84 ± 0.086^A^	0.59 ± 0.048^B^
Crypt, mm	0.28 ± 0.034	0.33 ± 0.039
Wall, mm	0.049 ± 0.018^a^	0.038 ± 0.013^b^
Villus/crypt	3.12 ± 0.35^A^	1.78 ± 0.25^B^

**Notes.**

In each row, different uppercase letters (A, B) indicate highly significant differences (*P* < 0.01), while different lowercase letters (a, b) indicate significant differences (*P* < 0.05).

The characteristics of the cecal environment are shown in Table 3. The concentration of acetic acid was significantly reduced after diarrhea (*P* < 0.05), but the concentrations of propionic acid and butyric acid as well as the ratio of acetic acid/(propionic acid + butyric acid) were comparable to those in the healthy group (*P* > 0.05). Moreover, diarrhea led to markedly increased pH (*P* < 0.05) and NH_3_-N content (*P* < 0.01). Under normal conditions, propagating probiotics produce lactic acid and acetic acid, reducing the pH in the digestive tract and modulating the microenvironment to inhibit the growth of harmful bacteria.

**Table 3:** Comparison of cecal environment between healthy rabbits and those with diarrhea.

Variable	Healthy	Diarrhea
Acetic acid, mg/mL	0.93 ± 0.089^a^	0.85 ± 0.0081^b^
Propionic acid, mg/mL	0.062 ± 0.0082	0.061 ± 0.0086
Butyric acid, mg/mL	0.27 ± 0.053	0.24 ± 0.0088
Acetic acid/(propionic acid + butyric acid)	2.80 ± 0.76	2.82 ± 0.64
pH	6.34 ± 0.21^b^	6.67 ± 0.15^a^
NH_3_-N, mg/dL	26.58 ± 3.45^B^	36.64 ± 4.15^A^

**Notes.**

In each row, different uppercase letters (A, B) indicate highly significant differences (*P* < 0.01), while different lowercase letters (a, b) indicate significant differences (*P* < 0.05).

### Comparison of serum indicators between healthy rabbits and those with diarrhea

Changes in serum indicators after diarrhea were examined. As shown in Table 4, serum TP in rabbits with diarrhea was significantly lower than that in the healthy group (*P* < 0.05), whereas AKP, UN, TNF-*α*, and IL-6 were significantly higher in the diarrhea group than the healthy group (*P* < 0.05). Levels of GLU, IgG, IgM, and IL-2 were not significantly different between the two groups (*P* > 0.05). These results suggest that protein breakdown and consumption increases in diarrhea, thus impairing body development.

**Figure 4:**
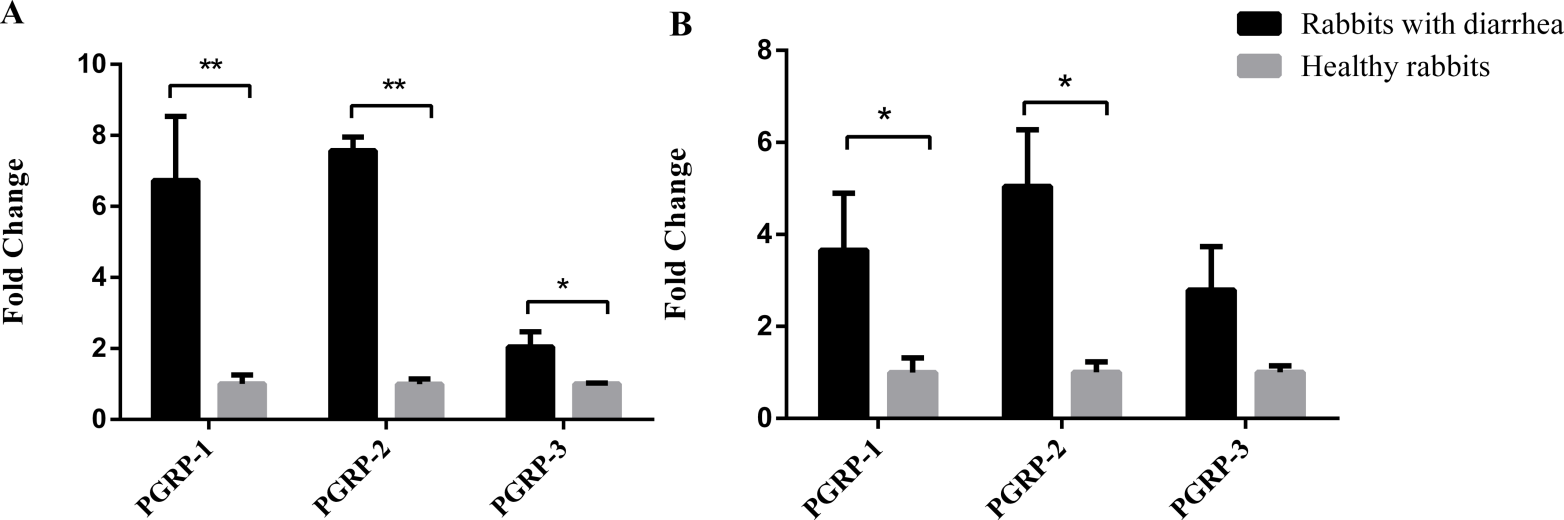
Diarrhea results in altered expression of PGRPs in the duodenum and cecum. Duodenum and cecum samples from three weaned rabbits with diarrhea and three healthy weaned rabbits were collected for real-time PCR. The results are presented as the mean ± SD at two levels of significance, *P < 0.05 and **P < 0.01. (A) Expression of PGRPs in the duodena of rabbits with diarrhea and healthy rabbits. (B) Expression of PGRPs in the ceca of rabbits with diarrhea and healthy rabbits.

**Table 4:** Comparison of immune indicators between healthy rabbits and those with diarrhea.

Indicator	Healthy	Diarrhea
TP, g/L	71.93 ± 8.86^a^	66.40 ± 7.53^b^
GLU, mmol/L	5.21 ± 0.57	5.01 ± 0.43
ALP, king unit/g	22.04 ± 5.04^b^	24.70 ± 5.21^a^
UN, mmol/L	7.96 ± 1.75^b^	10.84 ± 4.30^a^
IgG, mg/mL	10.08 ± 1.4	8.67 ± 1.63
IgM, mg/mL	2.70 ± 0.36	2.71 ± 0.26
TNF-*α*, pg/mL	1989.12 ± 148.01^b^	2657.64 ± 142.61^a^
IL-2, ng/L	13.11 ± 1.76	13.18 ± 1.73
IL-6, ng/L	243.16 ± 24.35^b^	250.02 ± 20.14^a^

**Notes.**

In each row, different uppercase letters (A, B) indicate highly significant differences (*P* < 0.01), while different lowercase letters (a, b) indicate significant differences (*P* < 0.05).

### Expression profile of PGRPs in healthy rabbits and those with diarrhea

Compared with the healthy group, the expression of antibacterial PGRPs increased in the ceca and duodena of rabbits with diarrhea (Fig. 4). *PGLYRP-1* and *PGLYRP-2* were both significantly upregulated in the ceca and duodena of rabbits with diarrhea compared with levels in healthy rabbits (*P* < 0.05), while *PGLYRP-3* was significantly upregulated in the ceca of rabbits with diarrhea (*P* < 0.05) but not significantly altered in the duodena (*P* > 0.05). These data suggest that young rabbits might combat harmful, diarrhea-causing intestinal bacteria by upregulating intestinal PGRPs.

### Analysis of floral differences between healthy rabbits and those with diarrhea

High-throughput sequencing data indicated that 57,286 ± 6,637 (mean ± SD) tags were detected in each sample, with no significant difference between the healthy (57,127 ± 7,484, mean ± SD) and diarrhea groups (57,445 ± 7,352, mean ± SD) (*P* > 0.05). To assess microbial diversity, clustering of effective tags was performed on all samples by sorting tags with ≥97% identity into an OTU. There were 916 ± 28 and 880 ± 46 OTUs in the healthy group and the diarrhea group, respectively, implying a higher diversity of cecal flora in healthy New Zealand rabbits. However, floral diversity was not significantly different between the diarrhea and healthy groups (*P* > 0.05) (Table 5).

**Figure 5:**
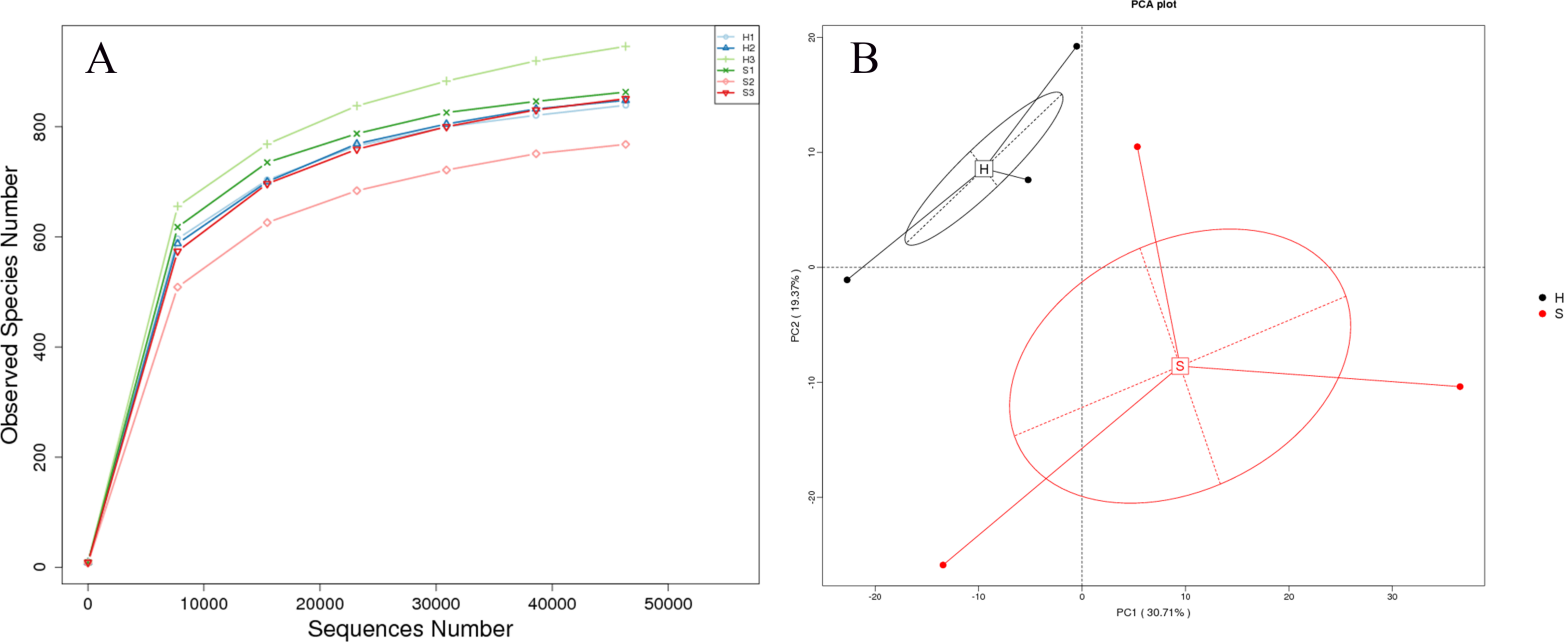
Rarefaction curves and PCA. (A) Rarefaction curves, reflecting sampling rationality, were generated from the OTU data. The rarefaction curve presents the relationship between sample number and OTU output. The plateau of the curve indicates a reduced number of new OTUs from increased sampling. (B) PCA extracts two axes that maximally reflect the variance among samples and present the variances from multi-dimensional data on a two-dimensional map, thereby revealing a simple pattern from complicated data. Shorter distances in the PCA plot indicate higher similarities of floral constitution between samples.

The rarefaction curve plateaued when the sequence number exceeded 6,000, and the OTU number did not change significantly with an increasing number of sequences thereafter. A minimum of 50,657 sequences were obtained from the tested samples, indicating that the sequence number was sufficient for analysis (Fig. 5A). PCA based on OTU levels (Fig. 5B) showed two prominent clusters from healthy and diarrhea samples, suggesting that healthy and diarrhea samples varied significantly.

**Table 5:** Number of tags and OTUs in rabbits with diarrhea and healthy rabbits.

Group	Total tags	Taxon tags	Unclassified tags	Unique tags	OTU number
Healthy	57,127 ± 7,484	51,464 ± 5,317	0	5,662 ± 2,352	916 ± 28
Diarrhea	57,445 ± 7,352	50,196 ± 3,315	0	7,248 ± 4,216	880 ± 46
*P*-value	0.961	0.744		0.600	0.302

Differences in the cecal floras of the diarrhea and healthy groups were analyzed at the phylum, class, order, family, genus, and species levels (Table 6). At the family level, the prevalences of three families of bacteria were significantly different between the healthy and diarrhea groups (*P* < 0.05), including *Planococcaceae*, which was 3.20-fold higher in the healthy group. Compared with healthy rabbits, five genera were significantly reduced in rabbits with diarrhea (*P* < 0.05), including *Clostridium* (2.38-fold lower), *Roseburia* (2.62-fold lower), and *Alistipes* (3.52-fold lower). At the species level, two species varied significantly between the healthy and diarrhea groups (*P* < 0.05), including *Alistipes indistinctus* (3.53-fold higher in healthy group) and *Coprococcus catus* (2.89-fold higher in the diarrhea group).

**Table 6:** Floral differences between healthy rabbits and those with diarrhea at different classification levels.

16S rRNA region/taxon						Abundance of bacterial taxa (%)		
Phylum	Class	Order	Family	Genus	Species	Healthy	Diarrhea	*P* value
Proteobacteria	Betaproteobacteria	Burkholderiales	Oxalobacteraceae			0.000907	0.000475	0.047818
Firmicutes	Bacilli	Lactobacillales	Enterococcaceae			0.000381	0.000237	0.027527
		Bacillales	Planococcaceae			0.000115	3.6E−05	0.0368
Firmicutes	Clostridia	Clostridiales	Lachnospiraceae	*Clostridium*		0.001561	0.000655	0.005818
				*Roseburia*		0.001	0.000381	0.004182
Proteobacteria	Betaproteobacteria	Burkholderiales	Oxalobacteraceae	*Oxalobacter*		0.000907	0.000475	0.036485
Bacteroidetes	Bacteroidia	Bacteroidales	Rikenellaceae	*Alistipes*		0.000583	0.000165	0.007455
Firmicutes	Bacilli	Lactobacillales	Enterococcaceae	*Enterococcus*		0.000381	0.000237	0.028773
Bacteroidetes	Bacteroidia	Bacteroidales	Rikenellaceae	*Alistipes*	*Indistinctus*	0.000583	0.000165	0.012083
Firmicutes	Clostridia	Clostridiales	Lachnospiraceae	*Coprococcus*	*Catus*	6.48E−05	0.000187	0.024208

## Discussion

Weaning induces psychological, environmental, and nutritional stress reactions in rabbits, which leads to diarrhea, dystrophia, and slow growth. In addition, the development of the digestive tracts of young rabbits is a complex and imperfect process. In the present study, we found that diarrhea in young rabbits was most prevalent at 30 days after weaning and caused intestinal dysfunction and changes in the internal environment. The intestinal microbial flora is known to be closely related to host health status. Drastic changes in microbial species and diversity in the intestinal mucosa may cause microbial imbalance and pathogenesis. Our results showed that weaned rabbits with diarrhea had shorter duodenal villi and a lower villus/crypt ratio, implying that diarrhea impaired intestinal development in weaned rabbits. A comparison of the cecal environment between healthy rabbits and those with diarrhea showed that the concentration of acetic acid decreased while NH_3_-N and pH increased in the ceca of rabbits with diarrhea. Factors related to the cecal environment, such as pH, VFA, and NH_3_-N, are known to be closely related to dynamic homeostasis (Buseth et al., 2015). The growth and reproduction of cecal bacteria require a suitable acidic environment, and VFA from food fermentation plays a critical role in the maintenance of an acidic environment in the intestines (Belenguer et al., 2011; Gidenne, 2013). The increase in alkaline NH_3_-N suggests an increased number of pathogens adapted to the alkaline environment (Gidenne & Jehl, 2000; Mourao et al., 2006).

Secreted PGRPs are known to exhibit antibacterial activities (Dziarski & Gupta, 2010; Liu et al., 2001). They do not stimulate innate immunity like the Toll-like receptors but instead directly recognize and kill bacteria. PGLYRP-2, for example, is a *N*-acetylmuramoyl-l-alanine amidase, and it can inhibit bacterial growth by degrading peptidoglycan (Gelius et al., 2003), whereas PGLYRP-1 and PGLYRP-3 kill bacteria directly (Dziarski et al., 2003; Lu et al., 2006; Wang et al., 2007a). In this study, the expression of PGRPs was upregulated in the ceca and duodena of rabbits with diarrhea. Moreover, serum biochemistry showed that diarrhea led to significantly reduced serum TP and increased serum AKP, UN, TNF-*α*, and IL-6 levels in young rabbits.

Furthermore, the intestinal floras of rabbits with diarrhea and healthy rabbits were analyzed by 16S rRNA V4 amplicon pyrosequencing. The highly conserved region was PCR-amplified using common primers, and the highly variable regions were sequenced for the identification of bacterial species. This technique is an important tool for characterizing microbial floras in environmental samples (Caporaso et al., 2011; Hess et al., 2011; Youssef et al., 2009). The results of the current study showed that the total counts and diversity of intestinal flora were comparable between healthy rabbits and those with diarrhea, but the frequencies of certain bacteria varied significantly, such as *Roseburia*, *Alistipes,* and *Clostridium*. *Roseburia* is a saccharolytic, butyrate-producing bacterium isolated from the cecum, and includes the species *Roseburia hominis*. The fecal microbiota of patients with inflammatory bowel disease contains a lower abundance of *R. hominis* than that of controls (Machiels et al., 2014). A significant decrease in *R. hominis* colonization was recently demonstrated in the guts of ulcerative colitis patients (Patterson et al., 2017). It has been suggested that the immunomodulatory properties of *Roseburia* could be useful for the control and treatment of gut inflammation in rabbit production as well. According to published reports, the genus *Clostridium* contains several species with different functions. Some of these can produce exotoxins, including *Clostridium difficile* and *Clostridium botulinum* (Jia et al., 2011). However, *Clostridium butyricum* promotes growth performance and immune function and improves the balance of the intestinal microflora in broiler chickens (Yang et al., 2012). We found that a lower abundance of some subtypes of *Clostridium* caused diarrhea, but the specific subtype involved was not clear and further experimental studies are required to confirm this finding. In addition, *Alistipes* was found to differ between healthy rabbits and those with diarrhea. Previous studies have shown that a greater frequency of abdominal pain is correlated with an increased abundance of several bacterial taxa from the genus *Alistipes* (Saulnier et al., 2011). *Alistipes* may be associated with abdominal pain in rabbits with diarrhea.

According to the findings of this study, weaned rabbits are prone to stress reactions, which cause diarrhea and inhibit growth. There are several underlying causes of this, including immune deficiency caused by weaning, an imbalance of the intestinal microflora, and other deleterious factors. Therefore, dietary supplementation of bacteria should be used to improve the composition of the intestinal flora of weaned rabbits. In addition, early feeding, slow weaning, and increased farm sanitation are recommended.

## Conclusions

In conclusion, diarrhea severely impairs intestinal development and immune function in young rabbits and induces the upregulation of intestinal antibacterial proteins. In addition, pyrosequencing of 16S rRNA V4 in the cecum indicated significant differences in the prevalences of *Clostridium, Roseburia*, and *Alistipes* between healthy rabbits and those with diarrhea, implying disruption of the microbiotas in the intestines of rabbits with diarrhea. Our findings provide evidence for an association between the intestinal microbial environment and host health in rabbits and contribute to a better understanding of the pathogenesis of diarrhea in young rabbits.

## Additional Information and Declarations

### Data Availability

The following information was supplied regarding data availability: 16S rDNA sequences were deposited with NCBI (accession number: SRP114741): https://trace.ncbi.nlm.nih.gov/Traces/sra/?study=SRP114741.

## References

[ref-1] Aerts JM, Boever JD, Maertens L (2010). Degradation of dietary oligofructose and inulin in the gastro-intestinal tract of the rabbit and the effects on caecal pH and volatile fatty acids. World Rabbitence.

[ref-2] Avershina E, Frisli T, Rudi K (2013). *De novo* semi-alignment of 16S rRNA gene sequences for deep phylogenetic characterization of next generation sequencing data. Microbes and Environments.

[ref-3] Belenguer A, Fondevila M, Balcells J, Abecia L, Lachica M, Carro MD (2011). Methanogenesis in rabbit caecum as affected by the fermentation pattern: *In vitro* and *in vivo* measurements. World Rabbit Science.

[ref-4] Beltz KM, Rosales MM, Morales E (2005). Histological and ultrastructural findings in commercial bred rabbits exhibiting severe diarrhea. Scandinavian Journal of Laboratory Animal Science.

[ref-5] Buseth ME, Saunders R, Buseth ME, Saunders R (2015). Rabbit behaviour, health and care.

[ref-6] Caporaso JG, Kuczynski J, Stombaugh J, Bittinger K, Bushman FD, Costello EK, Fierer N, Pena AG, Goodrich JK, Gordon JI, Huttley GA, Kelley ST, Knights D, Koenig JE, Ley RE, Lozupone CA, McDonald D, Muegge BD, Pirrung M, Reeder J, Sevinsky JR, Turnbaugh PJ, Walters WA, Widmann J, Yatsunenko T, Zaneveld J, Knight R (2010). QIIME allows analysis of high-throughput community sequencing data. Nature Methods.

[ref-7] Caporaso JG, Lauber CL, Walters WA, Berglyons D, Lozupone CA, Turnbaugh PJ, Fierer N, Knight R (2011). Global patterns of 16S rRNA diversity at a depth of millions of sequences per sample. Proceedings of the National Academy of Sciences of the United States of America.

[ref-8] De Blas C, Wiseman J (2010). Nutrition of the rabbit.

[ref-9] Dziarski R, Gupta D (2010). Review: mammalian peptidoglycan recognition proteins (PGRPs) in innate immunity. Innate Immunity.

[ref-10] Dziarski R, Platt KA, Gelius E, Steiner H, Gupta D (2003). Defect in neutrophil killing and increased susceptibility to infection with nonpathogenic gram-positive bacteria in peptidoglycan recognition protein-S (PGRP-S)-deficient mice. Blood.

[ref-11] Falk PG, Hooper LV, Midtvedt T, Gordon JI (1998). Creating and maintaining the gastrointestinal ecosystem: what we know and need to know from gnotobiology. Microbiology and Molecular Biology Reviews.

[ref-12] Gelius E, Persson C, Karlsson J, Steiner H (2003). A mammalian peptidoglycan recognition protein with N-acetylmuramoyl-L-alanine amidase activity. Biochemical & Biophysical Research Communications.

[ref-13] Gidenne T (2013). Dietary fibres: their analysis in animal feeding, and their role in rabbit nutrition and health. Indonesian Bulletin of Animal & Veterinary Sciences.

[ref-14] Gidenne T, Jehl N (2000). Caecal microbial activity of the young rabbit: incidence of a fibre deficiency and of feed intake level. World Rabbit Congress.

[ref-15] Guarner F, Malagelada JR (2003). Gut flora in health and disease. Lancet.

[ref-16] Hess M, Sczyrba A, Egan R, Kim TW, Chokhawala H, Schroth G, Luo S, Clark DS, Chen F, Zhang T (2011). Metagenomic discovery of biomass-degrading genes and genomes from cow rumen. Science.

[ref-17] Jia K, Zhu Y, Zhang Y, Li Y (2011). Group II intron-anchored gene deletion in *Clostridium*. PLOS ONE.

[ref-18] Kang D, Liu G, Lundström A, Gelius E, Steiner H (1998). A peptidoglycan recognition protein in innate immunity conserved from insects to humans. Proceedings of the National Academy of Sciences of the United States of America.

[ref-19] Liu C, Xu Z, Gupta D, Dziarski R (2001). Peptidoglycan recognition proteins: a novel family of four human innate immunity pattern recognition molecules. Journal of Biological Chemistry.

[ref-20] Lu X, Wang M, Qi J, Wang H, Li X, Gupta D, Dziarski R (2006). Peptidoglycan recognition proteins are a new class of human bactericidal proteins. Journal of Biological Chemistry.

[ref-21] Macfarlane S, Hopkins MJ, Macfarlane GT (2000). Bacterial growth and metabolism on surfaces in the large intestine. Microbial Ecology in Health & Disease.

[ref-22] Machiels K, Joossens M, Sabino J, De Preter V, Arijs I, Eeckhaut V, Ballet V, Claes K, Van Immerseel F, Verbeke K, Ferrante M, Verhaegen J, Rutgeerts P, Vermeire S (2014). A decrease of the butyrate-producing species *Roseburia hominis* and Faecalibacterium prausnitzii defines dysbiosis in patients with ulcerative colitis. Gut.

[ref-23] Magoc T, Salzberg SL (2011). FLASH: fast length adjustment of short reads to improve genome assemblies. Bioinformatics.

[ref-24] Mourao JL, Pinheiro V, Alves A, Guedes CM, Pinto L, Saavedra MJ, Spring P, Kocher A (2006). Effect of mannan oligosaccharides on the performance, intestinal morphology and cecal fermentation of fattening rabbits. Animal Feed Science & Technology.

[ref-25] Pascual JJ (2001). Early weaning of young rabbits: a review. World Rabbit Science.

[ref-26] Patterson AM, Mulder IE, Travis AJ, Lan A, Cerf-Bensussan N, Gaboriau-Routhiau V, Garden K, Logan E, Delday MI, Coutts AGP, Monnais E, Ferraria VC, Inoue R, Grant G, Aminov RI (2017). Human gut symbiont *Roseburia hominis* promotes and regulates innate immunity. Frontiers in Immunology.

[ref-27] Saulnier DM, Riehle K, Mistretta TA, Diaz MA, Mandal D, Raza S, Weidler EM, Qin X, Coarfa C, Milosavljevic A, Petrosino JF, Highlander S, Gibbs R, Lynch SV, Shulman RJ, Versalovic J (2011). Gastrointestinal microbiome signatures of pediatric patients with irritable bowel syndrome. Gastroenterology.

[ref-28] Wang Q, Garrity GM, Tiedje JM, Cole JR (2007b). Naive Bayesian classifier for rapid assignment of rRNA sequences into the new bacterial taxonomy. Applied and Environmental Microbiology.

[ref-29] Wang M, Liu LH, Wang S, Li X, Lu X, Gupta D, Dziarski R (2007a). Human peptidoglycan recognition proteins require zinc to kill both gram-positive and gram-negative bacteria and are synergistic with antibacterial peptides. Journal of Immunology.

[ref-30] Yang CM, Cao GT, Ferket PR, Liu TT, Zhou L, Zhang L, Xiao YP, Chen AG (2012). Effects of probiotic, *Clostridium butyricum*, on growth performance, immune function, and cecal microflora in broiler chickens. Poultry Science.

[ref-31] Youssef N, Sheik CS, Krumholz LR, Najar FZ, Roe BA, Elshahed MS (2009). Comparison of species richness estimates obtained using nearly complete fragments and simulated pyrosequencing-generated fragments in 16S rRNA gene-based environmental surveys. Applied & Environmental Microbiology.

